# SAK-HV Decreases the Self-Ubiquitination of MEKK1 to Promote Macrophage Proliferation via MAPK/ERK and JNK Pathways

**DOI:** 10.3390/ijms18040835

**Published:** 2017-04-19

**Authors:** Chao Zhang, Yao Chen, Xiangdong Gan, Zhiguang Huang, Minji Zou, Wenliang Fu, Weiwei Xing, Donggang Xu

**Affiliations:** The Laboratory of Genomic Engineering, Beijing Institute of Basic Medical Sciences, Beijing 100005, China; zhangchao0103@163.com (C.Z.); chenyao2014@163.com (Y.C.); gxd200837@163.com (X.G.); hzgdtd@163.com (Z.H.); zoumj@bmi.ac.cn (M.Z.); fwl86@139.com (W.F.); huozinangua@163.com (W.X.)

**Keywords:** macrophage proliferation, ubiquitination, Uba1, MEKK1, ERK, JNK

## Abstract

SAK-HV is an anti-atherosclerosis recombinant fusion protein developed by our lab. Our study determined that SAK-HV promoted macrophage proliferation, of which the mechanism was explored by both RAW264.7 cells and primary macrophages. Mass spectrometric analysis and co-immunoprecipitation were combined to screen the SAK-HV-interacting proteins in RAW264.7 cells. Confocal microscopy was adopted to detect the localization of SAK-HV in cells. The results indicated that SAK-HV triggered macrophage proliferation via the mitogen-activated protein kinase (MAPK)/extracellular signal-regulated kinases (ERK) and c-Jun N-terminal kinases (JNK) pathways by its SAK-mutant functional domain. We screened out Uba1 as the SAK-HV-interacting protein in the RAW264.7 cells and discovered their co-localization in the cytoplasm and nucleus. Inhibiting Uba1 significantly decreased the SAK-HV-induced macrophage proliferation. Thus, we postulated an attractive model of ubiquitination, in which the interactions between Uba1 and specific E2 enzymes are blocked by its interaction with SAK-HV. Based on this model, we detected the decreased self-ubiquitination of MEKK1 after SAK-HV treatment and concluded that SAK-HV inhibits the self-ubiquitination of MEKK1 via its SAK-mutant functional domain to activate MAPK/ERK and JNK pathways, promoting macrophage proliferation. This conclusion highly supported our hypothesized model of ubiquitination at the level of Uba1, which may represent a novel paradigm to promote macrophage proliferation by using the E1 enzyme (Uba1) as a switch.

## 1. Introduction

Macrophages are found in all tissues and play important roles in development, homeostasis, tissue repair, and immunity [1]. Traditionally, it was thought that tissue macrophages originated from the infiltration and differentiation of circulating monocytes [2]. However, an increasing number of studies have shown that the self-maintenance and self-renewal of many macrophages are more dependent on local proliferation [3,4,5]. Macrophage proliferation plays a key role in the development of metabolic syndrome. During the early phases of obesity, the accumulation of macrophages in adipose tissue is mainly dependent on in situ proliferation and causes positive protective effects, such as increased glucose tolerance and reduced blood glucose [6,7]. In contrast, some studies have shown that during the development of atherosclerosis, macrophage turnover becomes increasingly dependent on local proliferation at the lesion [8,9]. Hence, the effects of macrophage proliferation could play an important role in the middle and late phases of atherosclerotic plaque formation [8,9]. Since the effects of macrophage proliferation in physiological and pathological states could be a double-edged sword, the regulatory mechanism of macrophage proliferation has become a focus in recent years.

Cellular proliferation and signaling pathways are closely linked. The mitogen-activated protein kinase (MAPK) signaling pathway is a key evolutionarily conserved pathway that transduces extracellular signals to intracellular responses [10]. The three downstream members of this signaling cascade—the extracellular signal-regulated kinases (ERKs), the c-Jun N-terminal kinases (JNKs), and the p38 MAPKs–play important regulatory roles in the processes of cellular proliferation, differentiation, and apoptosis [10,11,12]. Another pathway that is closely associated with cellular proliferation is the Phosphoinositide 3-kinase/AKT (PI3K/AKT) signaling cascade [13,14]. Studies have shown that the MAPK/ERK and PI3K/AKT pathways play important roles in macrophage proliferation [15,16].

At the same time, these proliferation-related signaling pathways are also regulated by ubiquitination [13,17,18]. Ubiquitination is mediated by an E1-E2-E3 enzymatic cascade, of which ubiquitin-like modifier-activating enzyme 1 (Uba1) acts as the E1 enzyme in the first step [19]. Uba1 is the main initiator of the activation, conjugation, and ligation of ubiquitin to target protein substrates [20]. It activates ubiquitin and transfers it to E2-conjugating enzymes in an ATP- and Mg^2+^-dependent reaction by providing a favorable conformation of the Uba1-ubiquitin complex [21]. Then, the E2-ubiquitin complex and the protein targeted for ubiquitination are subsequently brought together by E3 ligases, which facilitates the transfer of activated ubiquitin from the E2 enzyme to the target protein [19]. Focusing on the close relationship between ubiquitination and proliferation-related signaling pathways, many studies have revealed that Uba1 is inevitably linked to cellular proliferation processes [17,22,23].

**Scheme 1:**
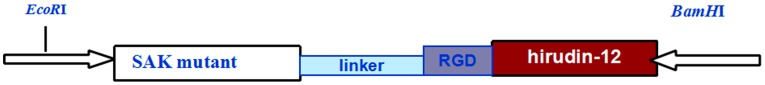
The domain map of SAK-HV: it was made of three functional domains: an SAK-mutated domain, an Arg-Gly-Asp (RGD) sequence, and a 12-amino acid peptide from the C-terminus of hirudin (hirudin-12).

We constructed a recombinant fusion protein (SAK-HV) that was made of three functional domains: an SAK-mutated domain, an Arg-Gly-Asp (RGD) sequence, and a 12-amino acid peptide from the C-terminus of hirudin (hirudin-12) (Scheme 1). Compared with wild-type SAK, SAK-mutant lost the first 10 amino acids. Besides, it also replaced the lysines 35 and lysines 136 with alanine, as well as inserting lysines 137. These mutations reduce both the molecular weight and the immunogenicity of SAK-HV, which was developed as a novel anti-atherosclerosis drug candidate, and has been shown to have multiple functions, including fibrinolysis triggered by SAK-mutant, anti-coagulation exerted by hirudin-12, and the inhibition of platelet aggregation induced by RGD [24]. Our previous study found that an injection of SAK-HV into *ApoE*^−/−^ mice could effectively lower inflammation and plasma lipid levels [25], as well as resulting in marked improvements in aortic plaque dispersion [26].

The liver is a central metabolic organ that can influence changes in plasma lipid levels, while aortic endothelial cells and macrophages are key cells that participate in the development of atherosclerosis. Therefore, we used SAK-HV to stimulate mouse aortic endothelial cells (MAEC), RAW264.7 murine macrophages, mouse embryonic liver cell line (BNL-CL2), and human acute monocytic leukemia cell line (THP-1), to screen for potential target cell lines. Unexpectedly, we found that SAK-HV stimulation could selectively induce proliferative effects in RAW264.7 macrophages, THP-1 monocytes, and primary peritoneal macrophage cells; however, this activity was not observed in the other cell lines. Therefore, we used both the RAW264.7 cell line and primary macrophages to investigate the mechanism of macrophage proliferation promoted by SAK-HV.

**Scheme 2:**
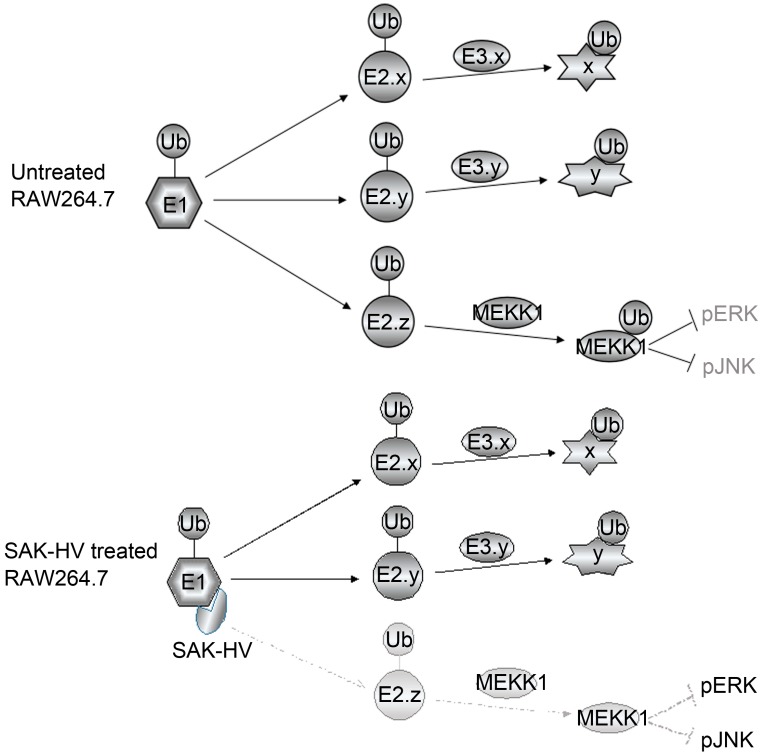
Schematic diagram of the hypothesized model of ubiquitination at the level of Uba1: Entry of SAK-HV into the RAW264.7 cells could partially inhibit the ubiquitination function of Uba1 through the interaction of its SAK-mutant functional domain and Uba1. This inhibition reduces the level of self-ubiquitination of MEKK1 by reducing the interaction between Uba1 and particular E2 enzymes to increase its kinase activity, which activates the MAPK/ERK and JNK pathways through downstream phosphorylation. Finally, macrophage proliferation is induced at the output of these cascades. The gray dotted line represents the blocked regulation between upstream and down stream. The gray pathway names indicates that these pathways are inactivated. Abbreviation: Ub, ubiquitin; pERK, phosphorylation of ERK pathway; pJNK, phosphorylation of JNK pathway.

In summary, our study illustrated that SAK-HV promotes macrophage proliferation through its SAK-mutant functional domain via MAPK/ERK and JNK pathways activated by the decreased MEKK1 self-ubiquitination. This might be explained by the interaction between Uba1 and SAK-HV, which decreased the self-ubiquitination of MEKK1, activating these two proliferation pathways (Scheme 2).

## 2. Results

### 2.1. SAK-HV Promotes the Proliferation of RAW264.7 Murine Macrophages

**Figure 1:**
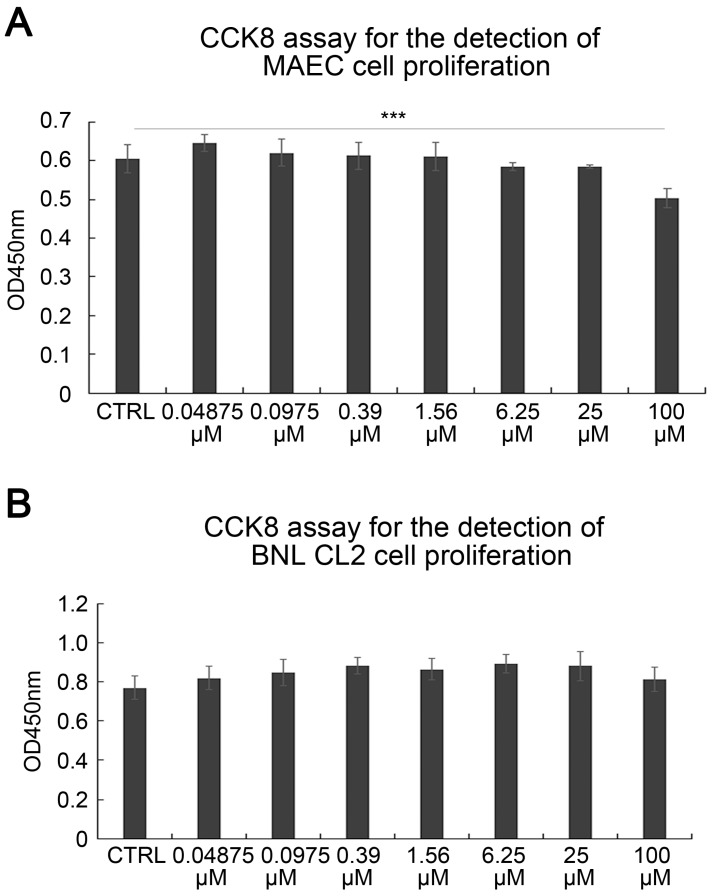
SAK-HV treatment did not induce proliferation in mouse aortic endothelial cells (MAEC) and BNL-CL2 mouse embryonic liver cells (*** *p* < 0.001). (**A**) mouse aortic endothelial cells (MAEC; *n* = 5); (**B**) BNL-CL2 mouse embryonic liver cells (*n* = 5). The MAEC and BNL CL2 cells were treated with increasing concentrations of SAK-HV (0, 0.04875, 0.0975, 0.39, 1.56, 6.25, 25, and 100 μM) for 24 h to investigate their proliferation effects via the CCK8 assay, separatively. Abbreviation: CTRL, control.

**Figure 2:**
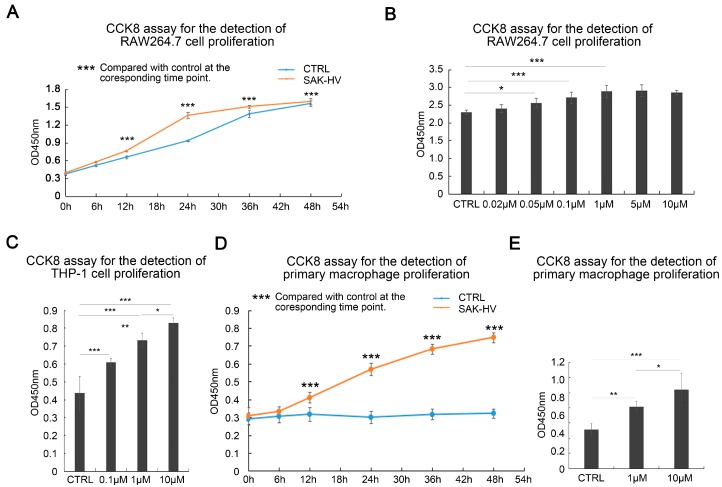
SAK-HV treatment significantly induced macrophage proliferation (* *p* < 0.05; ** *p* < 0.01; *** *p* < 0.001). (**A**) SAK-HV treatment induced proliferation in RAW264.7 cells in a time-dependent manner (*n* = 7); (**B**) SAK-HV treatment induced proliferation in RAW264.7 cells in a concentration-dependent manner (*n* = 5); (**C**) SAK-HV treatment induced proliferation in THP-1 cells in a concentration-dependent manner (*n* = 5); (**D**,**E**) SAK-HV treatment induced proliferation in primary macrophage cells (*n* = 6–7); The RAW264.7, THP-1 and primary macrophage cells were separatively treated with increasing concentrations of SAK-HV for 24 h to determine their proliferation effects via the CCK8 assays. RAW264.7 and primary macrophage cells were then treated with SAK-HV (1 μM for RAW264.7; 10 μM for primary macrophages) at multiple time points (0, 6, 12, 24, 36, and 48 h).

Increasing concentrations of SAK-HV were used to stimulate MAEC, RAW264.7 murine macrophages, and BNL-CL2 mouse embryonic liver cells for 24 h, to investigate the effects of SAK-HV on the degree of cellular proliferation of various cell types. The results showed that SAK-HV did not promote proliferation in MAEC and BNL-CL2 cells (Figure 1), but significantly increased cellular proliferation in RAW264.7 macrophages in a concentration- and time-dependent manner (Figure 2A,B). Similarly, the cell numbers of THP-1 human monocytes after SAK-HV treatment were significantly more than that of the control group, and were observed to be positively related to SAK-HV concentrations (Figure 2C). Considering that THP-1 cells are an actively dividing cell line, this result suggested that SAK-HV has a similar concentration-dependent proliferative effect in THP-1 human monocytes (Figure 2C). Furthermore, SAK-HV also induced the proliferation of primary peritoneal macrophage cells from C57BL/6J mice, which are normally non-dividing cells (Figure 2D,E). As a result, these results indicated that macrophages were one of the potential target cells of SAK-HV. We selected 1 μM as the subsequent SAK-HV treatment concentration and used both RAW264.7 and primary peritoneal macrophage cells to further investigate the mechanism by which SAK-HV induced proliferation in macrophages.

### 2.2. Effects of SAK-HV on Proliferation-Related Pathways in RAW264.7 Cells

**Figure 3:**
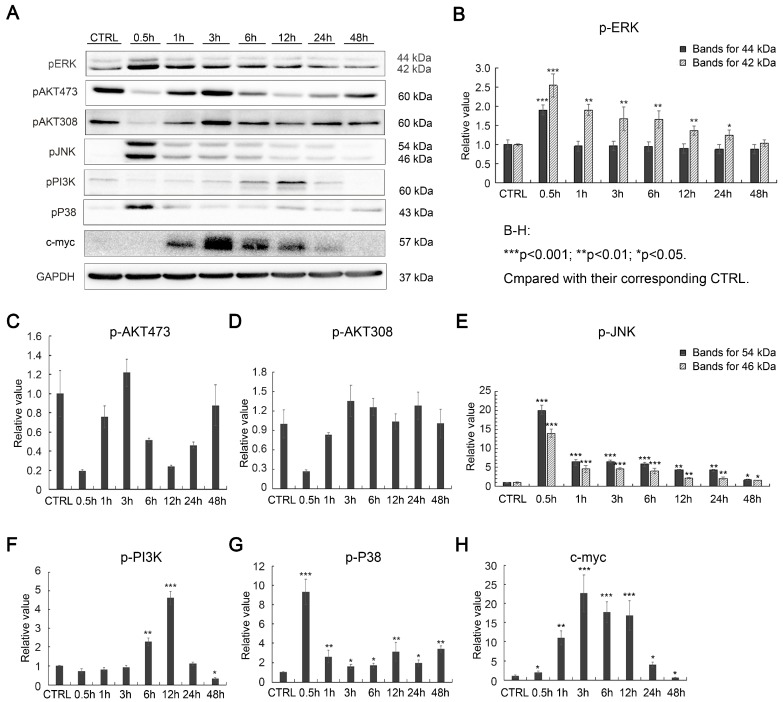
Effects of SAK-HV treatment on proliferation-related pathways (p38 MAPK, ERK, JNK, and PI3K/AKT) and the proliferation effector protein, c-Myc, in RAW264.7 cells at multiple time points (* *p* < 0.05; ** *p* < 0.01; *** *p* < 0.001). (**A**) The Western blot analysis for proliferation-related pathways (*n* = 3); (**B**–**H**) The bands quantification for Western blot analysis above. The RAW264.7 cells were treated with SAK-HV (1 μM) to determine its effects on the proliferation-related pathways via Western blot at multiple time points (0, 0.5, 1, 3, 6, 12, 24, and 48 h).

MAPK/ERK, JNK, p38 MAPK, and PI3K/AKT are pathways that were reported to be linked to cellular proliferation. We used multiple time points of the SAK-HV stimulation of RAW264.7 cells and examined the effects of SAK-HV on the ERK, JNK, p38 MAPK, and PI3K/AKT pathways through western blotting analyses. The results showed that p38 MAPK, JNK, and ERK activation all increased after 30 min of treatment and this was maintained until 48 h post-treatment, whereas the PI3K pathway was activated from 6 to 12 h post-treatment (Figure 3). AKT phosphorylations at residues 473 and 308 were inhibited from 30 min and gradually recovered at 3 h post-treatment, before cycling to be inhibited again and returning to their original state at 48 h post-treatment (Figure 3). The expression levels of the proliferation marker c-Myc began to increase at 1 h (Figure 3). From the above experiments, we excluded the possibility that SAK-HV exerts its proliferative effects through the activation of the AKT pathway, and showed that the activation time points of the p38 MAPK, JNK, and ERK pathways were more closely associated with the proliferation marker c-Myc than with the PI3K pathway.

**Figure 4:**
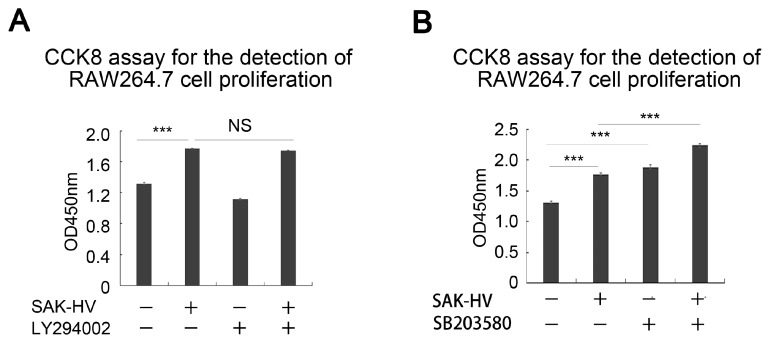
SAK-HV did not promote macrophage proliferation via PI3K or p38 MAPK pathways (*** *p* < 0.001). (**A**) The effect of inhibiting the PI3K pathway on the SAK-HV-triggered RAW264.7 cell proliferation (*n* = 4); (**B**) The effect of inhibiting the p38 MAPK pathway on the SAK-HV-triggered RAW264.7 cell proliferation (*n* = 4). RAW264.7 cells were treated with the PI3K inhibitor LY294002 (5 μM) and the p38 MAPK inhibitor SB203580 (10 μM) to determine the effects of these two pathways on the SAK-HV-mediated proliferation, respectively. Cells were treated with inhibitors for 1 h prior to SAK-HV stimulation.

**Figure 5:**
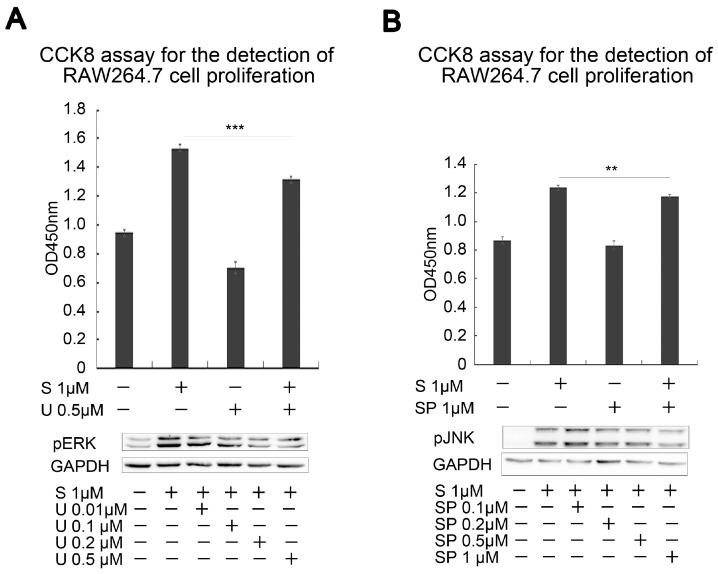
SAK-HV promoted macrophage proliferation via MAPK/ERK and JNK pathways (** *p* < 0.01; *** *p* < 0.001). (**A**) The effects of inhibitor U0126 at multiple concentrations on the phosphorylation level of ERK and the effect of inhibiting the MAPK/ERK pathway on the SAK-HV-triggered RAW264.7 cell proliferation (*n* = 4); (**B**) The effects of inhibitor SP600125 at multiple concentrations on the phosphorylation level of JNK and the effect of inhibiting the MAPK/JNK pathway on the SAK-HV-triggered RAW264.7 cell proliferation (*n* = 4). RAW264.7 cells were treated with the ERK inhibitor U0126 (0.01, 0.1, 0.2, and 0.5 μM) or the JNK inhibitor SP600125 (0.1, 0.2, 0.5, and 1 μM) to determine the effects of these inhibitors on these two pathways, separatively. The concentrations of 0.5 and 1 μM were selected as the U0126 and SB203580 treatment concentrations, respectively, for the CCK8 assays to determine SAK-HV-mediated proliferation. Cells were treated with inhibitors for 1 h prior to SAK-HV stimulation. Abbreviation: S, SAK-HV; U, U0126; SP, SP600125.

**Figure 6:**
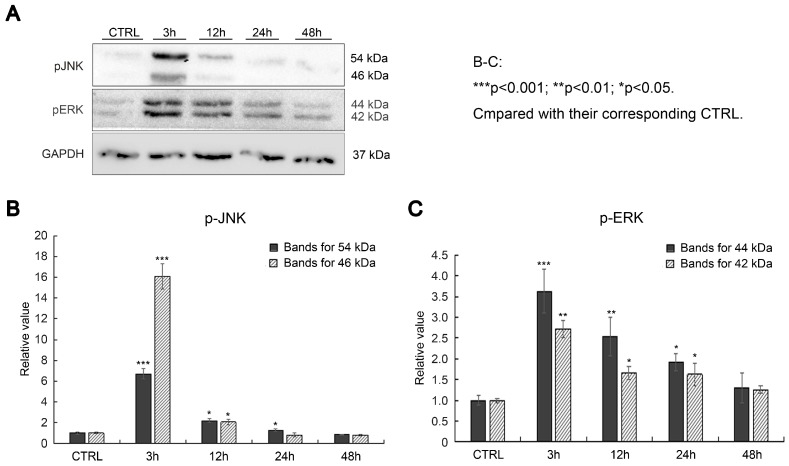
SAK-HV activated MAPK/ERK and JNK pathways in primary macrophages (* *p* < 0.05; ** *p* < 0.01; *** *p* < 0.001). (**A**) The Western blot for MAPK/ERK and JNK pathways in primary macrophages (*n* = 3); (**B**–**C**) The quantification of bands for Western blot. The primary macrophages were treated with SAK-HV (10 μM) to determine its effects on the proliferation-related pathways via Western blot at multiple time points (0, 3, 12, 24, and 48 h).

Furthermore, we inhibited the activated pathways to investigate their effects on RAW264.7 cell proliferation. The results showed that the PI3K inhibitor LY294002 had no significant effects on SAK-HV-induced proliferation (Figure 4A). On the other hand, the ERK inhibitor U0126 and the JNK inhibitor SP600125 showed inhibitory effects on RAW264.7 cell proliferation in the absence of SAK-HV treatment. We then screened different concentrations of these two inhibitors to investigate their inhibitory effects following SAK-HV stimulation for 30 min (Figure 5). During the screening, we selected for inhibitor concentrations that showed the closest inhibitory effects to the control group, while avoiding excessive inhibition in order to check the antagonism between SAK-HV and these inhibitors. The selected inhibitor concentrations were used to investigate the effects of ERK and JNK activation on SAK-HV-induced proliferation. The results showed that inhibitors of both the ERK and JNK pathways could significantly inhibit SAK-HV-induced proliferation in RAW264.7 cells (Figure 5). Interestingly, the p38 inhibitor SB203580 further enhanced SAK-HV-induced proliferation (Figure 4B). A previous study showed that SB203580 increases the phosphorylation levels of the ERK and JNK pathways [27]. This further verified the key roles of the ERK and JNK pathways in the SAK-HV-induced proliferation of RAW264.7 cells. Besides, we also observed the activation of ERK and JNK pathways in primary peritoneal macrophage cells from C57BL/6J mice after SAK-HV treatment (Figure 6), suggesting that this proliferation mechanism might also apply to primary macrophages in natural conditions.

### 2.3. SAK-HV Promotes Proliferation through Its SAK-Mutant Functional Domain in RAW264.7 Cells

**Figure 7:**
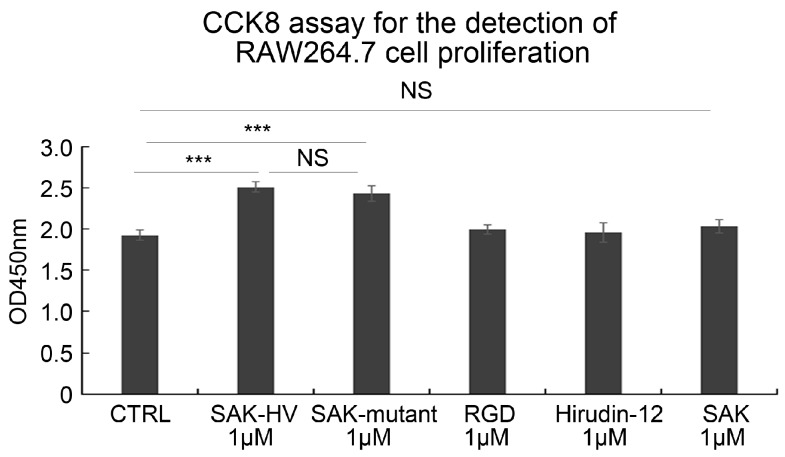
Structural analysis of SAK-HV-induced RAW264.7 cell proliferation (*** *p* < 0.001; NS, no significance). Among the three domains of SAK-HV—SAK-mutant, RGD, and hirudin-12—only the SAK-mutant domain could induce RAW264.7 cell proliferation at a level comparable to the full SAK-HV protein. Wild-type SAK had no effects on RAW264.7 cell proliferation (*n* = 6). RAW264.7 cells were treated with SAK-HV, the three individual functional domains of SAK-HV or the wild type SAK at 1 μM, to determine their effects on SAK-HV-mediated proliferation via the CCK8 assays. Abbreviation: hirudin-12, a 12-amino acid peptide from the C-terminus of hirudin.

We investigated whether SAK-HV exerts its macrophage proliferative effects through one of its specific functional domains. Using each of the functional domains individually, the results showed that the RGD domain and hirudin-12 did not induce the proliferation of RAW264.7 cells (Figure 7), while the SAK-mutant functional domain of SAK-HV effectively induced proliferation and was comparable to the level of proliferation induced by SAK-HV treatment. The SAK-mutant functional domain originated from the deletion mutation of the wild-type SAK protein to lower its immunogenicity [24]. Interestingly, the wild-type SAK had no effects on macrophage proliferation (Figure 7). These results showed that SAK-HV induces proliferation through its SAK-mutant functional domain.

### 2.4. Screening and Verification of SAK-HV-Interacting Proteins in RAW264.7 Cells

**Figure 8:**
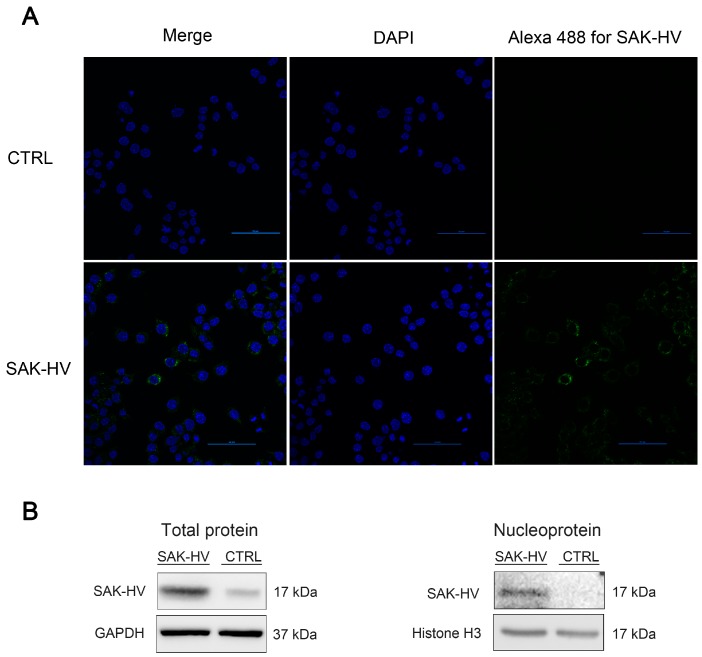
SAK-HV can enter into the cytoplasm and nucleus in RAW264.7 cells (Magnification, 600×; *n* = 4). (**A**) The distribution of SAK-HV in RAW264.7 cells; (**B**) The Western blot analysis for SAK-HV in RAW264.7 cell fractions (Left, total protein; Right, nucleoprotein).

**Figure 9:**
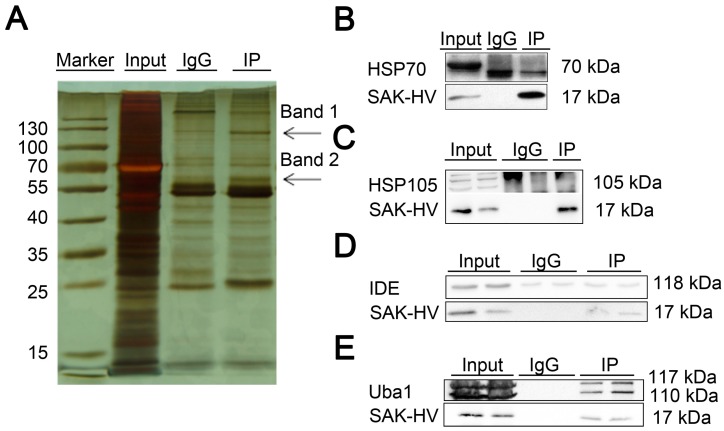
Screening of SAK-HV-interacting proteins in RAW264.7 cells: (**A**) Band 1 and Band 2 are the two selected polypeptide bands that were specifically bound to SAK-HV; (**B**–**E**) Immunoprecipitation identified Uba1 as a potential interacting protein of SAK-HV in RAW264.7 cells. Co-immunoprecipitation was adopted to identify the potential SAK-HV-interacting proteins by using SAK-HV-specific antibodies to capture proteins that were bound to SAK-HV. Then, the co-immunoprecipitated proteins were detected using antibodies against HSP70 for (**B**), HSP105 for (**C**), IDE for (**D**), and Uba1 for (**E**), among which HSP105, IDE, and Uba1 are proteins with the highest mass spectrometry score from Band 1, and HSP70 is the protein with the highest mass spectrometry score from Band 2. Either the alteration of protein conformation or the protein degradation during treatment might exert some effects on the reduced level of Hsp70 (70 kDa) in the silver staining of acrylamide gel. However, the mass spectrometric analysis identified Hsp70 as the potential SAK-HV-interacting protein in Band 2 with the highest mass spectrometry score.

**Figure 10:**
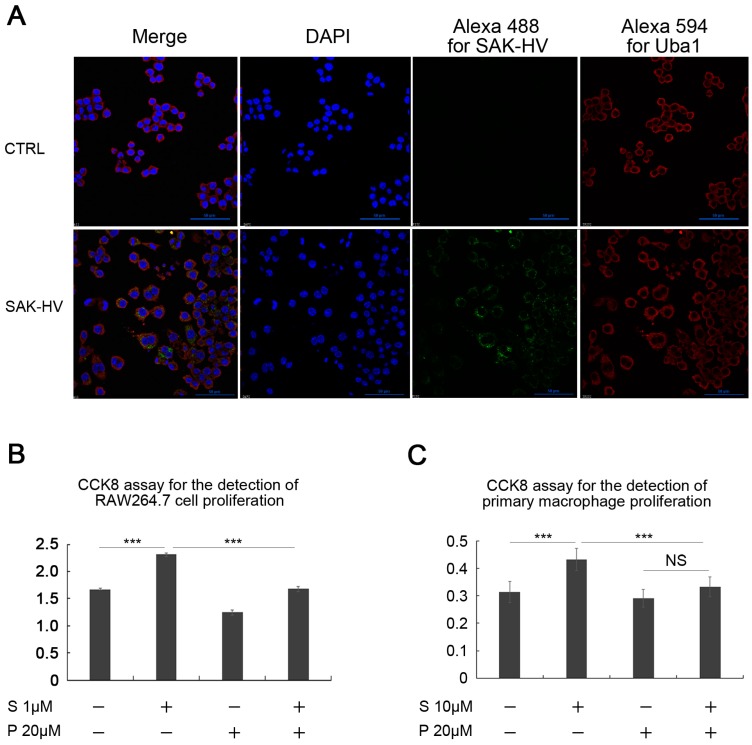
The important role of Uba1 in SAK-HV-mediated macrophage proliferation (*** *p* < 0.001; NS, no significance) (Magnification, 600×; *n* = 4). (**A**) Confocal microscopy images of the co-localization of SAK-HV and Uba1 in the cytoplasm and nucleus of RAW264.7 cells; (**B**,**C**) Uba1 inhibition significantly broke the proliferation effect of SAK-HV in RAW264.7 (*n* = 4) and primary macrophage cells (*n* = 6). Abbreviation: S, SAK-HV; P, PAR-41.

The use of confocal microscopy showed that SAK-HV could enter into the cytoplasm, and even the nucleus in smaller amounts (Figure 8A), which was further supported by the Western blot analysis of RAW264.7 cell fractions (Figure 8B), suggesting that SAK-HV might have interacting proteins within the macrophages. Through the silver staining of acrylamide gels of RAW264.7 extracts, we selected two polypeptide bands that were specifically bound to SAK-HV (Figure 9A). Through mass spectrometric analysis, we screened for potential interacting proteins of SAK-HV and used co-immunoprecipitation to further verify the proteins with the highest mass spectrometry scores (Table 1). The results showed that Uba1 directly interacted with SAK-HV (Figure 9B–E). At the same time, confocal microscopy showed that both proteins were co-localized in the cytoplasm and within the nucleus of the RAW264.7 cells (Figure 10A). These results suggested that SAK-HV could interact with Uba1 to activate the ERK and JNK pathways, thereby inducing macrophage proliferation.

We further adopt the Uba1 inhibitor PAR-41 to examine the role of Uba1 in SAK-HV-enhanced macrophage proliferation. The SAK-HV-enhanced proliferation effects in both the RAW264.7 and primary macrophage cells were remarkably ameliorated by PAR-41 (Figure 10B,C). These results confirmed the critical role of Uba1 in macrophage proliferation induced by SAK-HV.

### 2.5. SAK-HV Inhibited the Self-Ubiquitination of the Ubiquitin Ligase MAPK/ERK Kinase Kinase 1 (MEKK1) to Activate both ERK and JNK Pathways

**Figure 11:**
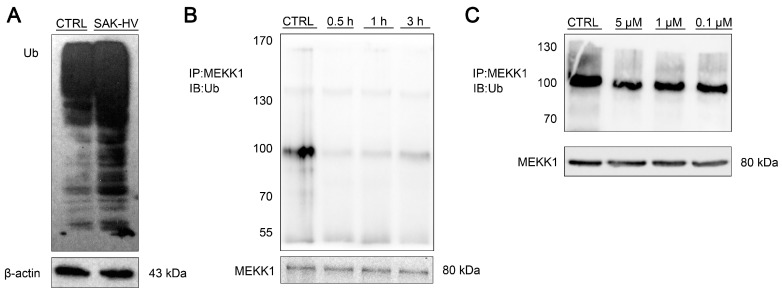
SAK-HV treatment inhibited MEKK1-mediated self-ubiquitination: (**A**) SAK-HV treatment increased the overall ubiquitination levels in RAW264.7 cells; (**B**,**C**) SAK-HV treatment decreases the levels of self-ubiquitinated MEKK1 in RAW264.7 cells. Raw264.7 cells were treated with SAK-HV at 1 μM for multiple processing lengths (0, 0.5, 1, and 3 h) separatively in panel B. Raw264.7 cells were treated with SAK-HV for 0.5 h at multiple concentrations (0.1, 1, and 5 μM) separatively in panel **C**.

Uba1 is a central enzyme in the ubiquitin modification machinery. The entry of Uba1 into the nucleus can trigger cellular proliferation [28]. We used cNLS Mapper to analyze the SAK-HV sequence, but did not find any nuclear localization signals (NLS). Moreover, the self-ubiquitination of MEKK1 could inhibit its own kinase activity and affect the JNK and ERK/MAPK pathways by the inhibition of their phosphorylation [29]. Hence, we investigated the effects of SAK-HV on the MEKK1 ubiquitination level. The results showed that after SAK-HV treatment, the proliferation of RAW264.7 cells was accompanied by an overall increase in ubiquitination levels (Figure 11A), but the levels of ubiquitinated-MEKK1 were significantly decreased (Figure 11B,C). These results showed that SAK-HV decreased the levels of self-ubiquitinated MEKK1, resulting in an increase in the phosphorylation levels of JNK and ERK. The interaction between SAK-HV and Uba1 may play an important role in macrophage proliferation via the MEKK1-mediated ubiquitination pathway.

## 3. Discussion

Macrophage proliferation is involved in many biological processes and the mechanisms of initiating proliferation have become a focus in recent years [30,31,32]. We found that SAK-HV could selectively induce the macrophage proliferation, and explored its molecular mechanism using RAW264.7 cells as a model. Our study illustrated the mechanism of SAK-HV-promoted macrophage proliferation, in which Uba1 might play an important role.

The screening of multiple proliferation-related pathways showed that SAK-HV treatment inhibited AKT signaling in a phasic manner, while activating the PI3K, MAPK p38, JNK, and ERK pathways. Further experiments with inhibitors of the relevant pathways verified that SAK-HV induced proliferation in RAW264.7 cells through the ERK and JNK pathways. Moreover, of the three functional domains of SAK-HV, only the SAK-mutant functional domain increased the proliferation of RAW264.7 cells, with intensities comparable to those of the full SAK-HV protein treatment. Interestingly, wild-type SAK did not induce the proliferation of RAW264.7 cells. These results indicated that SAK-HV promoted macrophage proliferation by the JNK and ERK/MAPK pathways via its SAK-mutant functional domain.

How does SAK-HV activate the JNK and ERK/MAPK pathways to promote macrophage proliferation? We further proved that SAK-HV could enter the cytoplasm and nucleus, suggesting potential intracellular SAK-HV-interacting proteins in RAW264.7 cells. We used mass spectrometry and co-immunoprecipitation to identify Uba1 as the interacting protein of SAK-HV in RAW264.7 cells. Confocal microscopy analysis showed they were co-localized in the cytoplasm and nucleus, further supporting that they could interact with one another. By Uba1 inhibition in both RAW264.7 and primary macrophage cells, we further verified the important role of Uba1 in macrophage proliferation induced by SAK-HV. These results strongly supported the idea that SAK-HV could induce macrophage proliferation through its interaction with Uba1.

Uba1 is the first enzyme in the ubiquitination process and is closely related to the regulation of cellular proliferation. Uba1 participates in the regulation of nuclear RNA replication, where defects in Uba1 can cause cell cycle arrest at the synthesis (S)/second gap (G2) or G2 phase in many cell types [33,34,35,36,37]. Uba1 enters the nucleus to promote proliferation and is phosphorylated in a cell cycle-dependent manner, during which its expression levels significantly increase, compared to its levels during the resting state [17,38]. On the other hand, multiple proliferation pathways are under the control of the Uba1-mediated regulation of ubiquitination, which occurs as a specific pair of E2 and E3 enzymes targets a particular protein. Therefore, the interaction between SAK-HV and Uba1 could induce cell proliferation through two possible mechanisms: (1) SAK-HV acts to increase Uba1’s nuclear entry or by increasing Uba1’s level of phosphorylation to trigger macrophage cell cycle progression [17,38,39,40]; or (2) SAK-HV reduces Uba1’s interaction with a specific pair of E2 and E3 enzymes that targets particular pathways, thereby promoting macrophage proliferation.

Our study showed that the first proposed mechanism seems implausible. The nuclear entry of Uba1 requires a NLS sequence and Uba1 phosphorylation could enhance nuclear targeting and retention [38,39]. The sequence analysis of SAK-HV did not find any NLS sequences, excluding the possibility of SAK-HV increasing Uba1 nuclear entry. We could foresee an increase in nuclear Uba1 and its phosphorylation after SAK-HV treatment in RAW264.7 cells, since it promoted the proliferation of RAW264.7 cells. However, it is difficult to determine whether this change is a cause or an effect of SAK-HV-induced proliferation. On the other hand, the entry of Uba1 into the nucleus and the increase in its phosphorylation would trigger proliferation responses in multiple cell types [40,41,42], so it could be a common proliferative mechanism. However, SAK-HV did not induce proliferation in aortic endothelial cells or in embryonic liver cells, further suggesting that it probably does not induce macrophage proliferation via the first proposed mechanism. Therefore, we proposed that SAK-HV would most probably use the second proposed mechanism: affecting the ubiquitin modification status of specific proliferation-related pathways to selectively induce macrophage proliferation. In other words, we established a hypothesized model of ubiquitination, in which the interactions between Uba1 and specific E2 enzymes are blocked by its interaction with SAK-HV.

We proved that ERK and JNK are crucial pathways by which SAK-HV promotes RAW264.7 cell proliferation. Through self-ubiquitination, MEKK1 could affect the downstream activity of both the ERK and JNK pathways [29]. Based on our hypothesized model, these results suggested that MEKK-mediated self-ubiquitination could be regulated by the interaction between SAK-HV and Uba1 to activate these two proliferation pathways of SAK-HV. As expected, following SAK-HV stimulation for 30 min, we detected that SAK-HV-enhanced macrophage proliferation was accompanied by an overall increase in the cellular ubiquitination levels, but the level of ubiquitinated-MEKK1 was significantly decreased. Unexpectedly, the desecrated self-ubiquitination of MEKK1 in the presence of different concentrations of SAK-HV was modest and seemed to occur in a dose-independent manner, so the detailed mechanism of MEKK1 self-ubiquitination in the presence and the absence of SAK-HV still needs further research. In summary, our study illustrated that SAK-HV decreased the self-ubiquitination of MEKK1 to activate the MAPK ERK and JNK pathways, thereby enhancing macrophage proliferation.

This conclusion strongly supported our hypothesized model that Uba1 might mediate the SAK-HV-induced cell proliferation via the second mechanism. That is, SAK-HV might interact with Uba1 to decrease MEKK1 self-ubiquitination by reducing the interaction between Uba1 and particular E2 enzymes, thereby activating the MAPK ERK and JNK pathways to enhance macrophage proliferation (Scheme 2). This novel mechanism reflects the forward-looking nature of our hypothesized model.

The regulation of ubiquitination was thought to occur at the level of a specific pair of E2 and E3 enzymes, which targets particular proteins, so our study suggested an attractive model for ubiquitination, in which E1 enzyme is involved. Interestingly, a study has hypothesized that the E1 enzyme could be involved in the regulation of ubiquitin modifications through changes in its phosphorylation levels, acting as a master switch of macrophage differentiation [43]. Similarly, our study suggested that Uba1 could participate in the regulation of ubiquitination under the influence of SAK-HV, acting as a switch of macrophage proliferation. Actually, SAK-HV not only enhanced the proliferation of actively dividing RAW264.7 and THP-1 cells, but also induced the proliferation of normally non-dividing primary macrophages. The activation of the two proliferation pathways of SAK-HV, ERK and JNK MAPK, in both RAW264.7 and primary macrophage cells, as well as the effective inhibitory effects of PAR-41 on SAK-HV-mediated proliferation in these two kinds of cells, highly supported that this novel model might also be applied to the terminally-differentiated macrophage proliferation in natural conditions.

## 4. Materials and Methods

### 4.1. Cell Culture

All cells were purchased from The National Experimental Cell Resource Sharing Platform, and were grown in 150-mm cell culture dishes with DMEM supplemented with 10% FBS, 100 U/mL penicillin (Gibco, Grand Island, NY, USA), and 100 mg/L streptomycin (Gibco) in a humidified incubator with 5% CO_2_ at 37 °C. Cells were incubated for 24 h prior to treatment.

### 4.2. CCK8 Assay

Cell viability and proliferation were measured using the Cell Counting Kit 8 (CCK8; Dojindo, Kumamoto, Japan) assay. Cells were collected and seeded into 96-well plates at a cell density of 2 × 10^4^ cells per well. After being cultured for 24 h, cells were treated as follows. For THP-1 cells, they were treated with SAK-HV (1 μM) at the 24th, 36th, and 42rd hour, or were untreated from start to finish. Then, the cell numbers were detected at the 48th hour. For RAW264.7 and primary macrophage cells, cells were treated with SAK-HV (1 μM for RAW264.7; 10 μM for primary macrophages) or were untreated as the control for various time lengths, including 0, 6, 12, 24, 36, and 48 h. They were also treated with SAK-HV at various concentrations or untreated as the control for 24 h. After the treatments, ten microliters of CCK8 assay solution was added to each well and incubated for 2 h. The absorbance was measured at 450 nm for each well using a multi-well spectrophotometer. Moreover, for the Uba1 inhibition assays, the inhibitor PAR-41 was added to the corresponding wells at the same time as the SAK-HV treatment.

### 4.3. Western Blot

Nuclear proteins were extracted by the Nuclear Extraction Kit (CWBIO, Beijing, China) according to the manufacturer’s instructions. The total proteins were isolated from cells using radio immunoprecipitation assay (RIPA) lysis buffer, and their concentrations were measured via the BCA assay (Thermo Fisher Scientific, Inc., Rockford, IL, USA). Equal amounts of protein per sample were electrophoresed through SDS-PAGE (12% polyacrylamide). The proteins were transferred onto a PVDF membrane via wet driving (Bio-Rad, Hercules, CA, USA). Blots were blocked for 1 h with 5% milk in 1× TBS-T (150 mmol/L NaCl; 50 mmol/L Tris; pH7.5; 0.02% Tween 20), followed by the overnight incubation with primary antibodies listed below, diluted in blocking buffer. The primary antibodies included p-ERK1/2 (Cell Signaling Technology, Boston, MA, USA), p-JNK (Cell Signaling Technology), p-PI3K (Tyr458) (Cell Signaling Technology), p-AKT (Ser473/Ser308) (Cell Signaling Technology), p-p38 (Sigma-Aldrich, Saint Louis, MO, USA), c-Myc (Santa Cruz Biotechnology, Heidelberg, Germany), MEKK1 (Santa Cruz Biotechnology), anti-SAK-HV (CWbio, Beijing, China), and GAPDH (CWbio). Then, the PVDF membrane was washed by TBS-T five times (5 min per time), and was incubated for 1 h with secondary HRP anti-rabbit IgG (Sigma-Aldrich). After having been washed with TBS-T five times (5 min per time), the immunoreactive bands were visualized with the ECL luminescence reagent (Pierce, Rockford, FL, USA). The immunoreactive bands were quantified using Quantity One software (version 4.62, Bio-Rad, Hercules, CA, USA).

### 4.4. Mass Spectrometric Analysis

For mass fingerprinting, the SDS-PAGE separated protein samples were detected by silver staining. Bands were sent to the National Center of Biomedical Analysis for mass spectrometric analysis by a Thermo Scientific Q Exactive mass spectrometer. In-gel digestions of the selected protein spots on the gels were performed as described in [44].

### 4.5. Confocal Microscopy

The RAW264.7 cells were planted onto a confocal dish at a density of 3 × 10^5^ cells per well. After being cultured in the medium for 24 h, the cells in the SAK-HV group were stimulated with SAK-HV for 24 h. Cells were then fixed with 4% paraformaldehyde in PBS for 15 min. Following three PBS washes, the cells were further fixed in methanol at −20 °C for 15 min. After three washes, the cells were blocked in 5% BSA/PBS (*v*/*v*) for 1 h at room temperature, then incubated with anti-SAK-HV (CWbio) or together with anti-UBA1 (Abcam plc, Cambridge, UK) antibodies in the blocking solution overnight at 4 °C. Following three washes, they were incubated with the Fluorescent-dye conjugated secondary antibodies (Alexa Fluor 488-conjugated anti-SAK-HV, and Alexa Fluor 594-conjugated anti-Uba1) for 1 h. After being incubated with DAPI (Solarbio, Beijing, China) for cell-nuclei staining for 5 min, these cells were examined using a Leica confocal laser scanning microscope (Leica, Wetzlar, Germany).

### 4.6. Co-Immunoprecipitation Assay

Protein extractions from cultured cells with a modified buffer from cultured cells were followed by immunoprecipitation and immunoblotting assays with the corresponding antibodies, as described in the following. The detection of SAK-HV was determined by immunoblotting using an antibody purchased from CWbio, while the co-immunoprecipitated proteins were detected using antibodies against Uba1 (Abcam plc), HSP105 (Abcam plc), HSP70 (Abcam plc), and IDE (Santa Cruz Biotechnology). Immunoprecipitated material was detected using the indicated primary antibody and HRP-conjugated anti-rabbit or anti-mouse IgG (Sigma-Aldrich).

### 4.7. Ubiquitination Assay

Raw264.7 cells were seeded in 60-mm cell culture dishes (Corning-Costar). For the time-response assay of MEKK1 ubiquitination treated by SAK-HV, cells were treated with SAK-HV at 1 μM for multiple processing lengths (0, 0.5, 1, and 3 h). For the dose-response assay of MEKK1 ubiquitination treated by SAK-HV, cells were treated with SAK-HV for 0.5 h at multiple concentrations (0.1, 1, 5 μM). After SAK-HV treatment, the whole-cell lysates were prepared in ice-cold RIPA buffer (CWbio). The protein content was determined by using the BCA protein assay (Pierce) after collecting the cell supernatant. A total of 500 μg of cellular proteins was transferred to 1.5-mL microcentrifuge tubes. These proteins were incubated at 4 °C overnight with 2 μg anti-MEKK1 (Santa Cruz Biotechnology, Heidelberg, Germany), together with 20 μL of Protein A/G PLUS-Agarose (Santa Cruz Biotechnology). After being washed four times with 1.0 mL RIPA buffer, the samples were boiled for 2–3 min to prepare the immunoprecipitated MEKK1 protein samples. For the MEKK1 immunoprecipitation of ubiquitination detection, cells were treated with 10 μM MG132 (American Peptide, Sunnyvale, CA, USA) for 90 min at 72 h post-treatment of SAK-HV. Half of the immunoprecipitates were loaded onto one gel. The gel was transferred onto the PVDF membrane, then prepared for Ubiquitin blotting. These samples were analyzed via Western blot to check the ubiquitination levels of the equal amounts of MEKK1 protein from the control group and all the SAK-HV treatment groups.

### 4.8. The Extraction of Primary Peritoneal Macrophage Cells from C57BL/6J Mice

**Figure 12:**
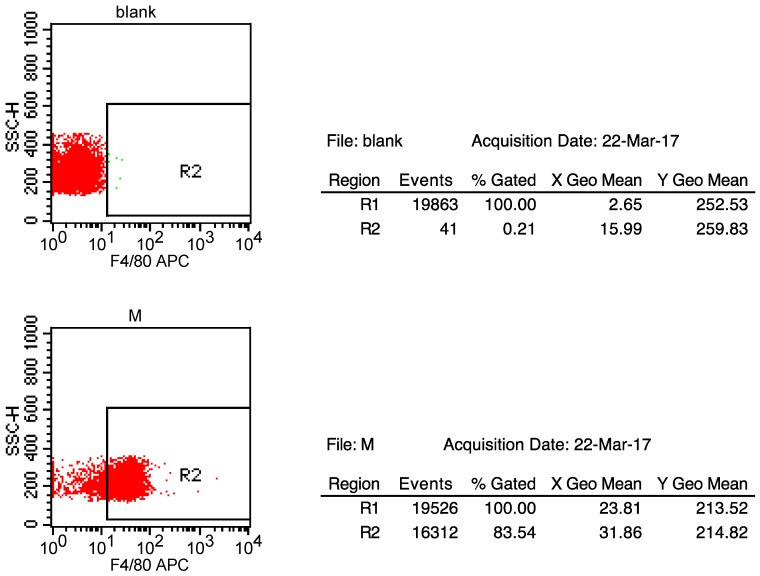
The separation of primary macrophages by F4/80 Antigen APC via flow cytometry. Abbreviation: M, macrophages.

Male C57BL/6J mice aged 6–8 weeks were immediately soaked in alcohol for about 5 min after being executed. Then, the abdominal skin of the mice was torn without breaking their peritoneal membrane. About 5 mL saline was injected into the abdominal cavity through syringes, and the saline in the abdominal cavity was well shaken. After having rested for about 5 min, the saline in the abdominal cavity was collected in sterile centrifuge tubes, and the cells were separated by a centrifuge (1200 r/min) for 5 min. Then, all cells were transferred into the cell culture dishes with DMEM supplemented with 10% FBS. Furthermore, these cells were incubated for 30 min with 20 μg/mL of APC-conjugated rat anti-mouse F4/80 (eBioscience, San Diego, CA, USA). Following this, the separation of primary macrophages from these cells by flow cytometry was performed by the National Center of Biomedical Analysis (Figure 12). The animal experiment was approved by the local government authorities and was carried out according to the guidelines of the Institutional Animal Care and Use Committee of the Academy of Military Medical Sciences (Approval number: IACUC of AMMS-13-2015-008).

### 4.9. Statistics

Statistical analyses were performed in R using one-way ANOVA or two-way ANOVA, both with the Tukey’s multiple comparisons post-hoc test and the unpaired 2-tailed Student’s *t* test, according to the experimental grouping designs. *p*-Values of ≤0.05 were considered statistically significant. Data in the figures were expressed as means ± SE.

## 5. Conclusions

In conclusion, our study proposed a novel paradigm of macrophage proliferation via a distinctive model of ubiquitination at the level of Uba1 (Scheme 2). Based on this model, we illustrated that SAK-HV decreased the self-ubiquitination of MEKK1 to enhance macrophage proliferation via the MAPK ERK and JNK pathways by its SAK-mutant functional domain, although this model requires further verification.

## Figures and Tables

**Table 1:** The potential SAK-HV-interacting proteins with the highest mass spectrometry scores.

No.	Name	Band	Score
gi|6678483	ubiquitin-like modifier-activating enzyme 1 isoform 1 (Uba1)	Band 1 ^1^	341
gi|114145505	heat shock protein 105 kDa (HSP105)	Band 1 ^1^	167
gi|14548072	nsulin-degrading enzyme (IDE)	Band 1 ^1^	148
gi|435839	heat shock protein 70 kDa (HSP70)	Band 2 ^1^	51

^1^ Band 1 and Band 2 are the two selected polypeptide bands that were specifically bound to SAK-HV in Figure 9A.

## Abbreviations

MAPK	mitogen-activated protein kinase
ERKs	extracellular signal-regulated kinases
JNKs	c-Jun N-terminal kinases
LD	ubiquitin-like modifier-activating enzyme 1
RGD	Arg-Gly-Asp
PI3K	Phosphoinositide 3-kinase
MAEC	mouse aortic endothelial cells
hirudin-12	12-amino acid peptide from the C-terminus of hirudin
MEKK1	MAPK/ERK kinase kinase 1
NLS	nuclear localization signal
